# Role of oxidative stress in neurodegenerative disorders: a review of reactive oxygen species and prevention by antioxidants

**DOI:** 10.1093/braincomms/fcad356

**Published:** 2024-01-02

**Authors:** Annwyne Houldsworth

**Affiliations:** University of Exeter Medical School, Exeter, Devon EX2 4TH, UK

**Keywords:** superoxide dismutase, reactive oxygen species, Alzheimer’s disease, neurodegeneration, avasopasem manganese

## Abstract

Neurological disorders include a variety of conditions, including Alzheimer’s disease, motor neuron disease and Parkinson’s disease, affecting longevity and quality of life, and their pathogenesis is associated with oxidative stress. Several of the chronic neurodegenerative pathologies of the CNS share some common features, such as oxidative stress, inflammation, synapse dysfunctions, protein misfolding and defective autophagia. Neuroinflammation can involve the activation of mast cells, contributing to oxidative stress, in addition to other sources of reactive oxygen species. Antioxidants can powerfully neutralize reactive oxygen species and free radicals, decreasing oxidative damage. Antioxidant genes, like the manganese superoxide dismutase enzyme, can undergo epigenetic changes that reduce their expression, thus increasing oxidative stress in tissue. Alternatively, DNA can be altered by free radical damage. The epigenetic landscape of these genes can change antioxidant function and may result in neurodegenerative disease. This imbalance of free radical production and antioxidant function increases the reactive oxygen species that cause cell damage in neurons and is often observed as an age-related event. Increased antioxidant expression in mice is protective against reactive oxygen species in neurons as is the exogenous supplementation of antioxidants. Manganese superoxide dismutase requires manganese for its enzymic function. Antioxidant therapy is considered for age-related neurodegenerative diseases, and a new mimetic of a manganese superoxide dismutase, avasopasem manganese, is described and suggested as a putative treatment to reduce the oxidative stress that causes neurodegenerative disease. The aim of this narrative review is to explore the evidence that oxidative stress causes neurodegenerative damage and the role of antioxidant genes in inhibiting reactive oxygen species damage. Can the neuronal environment of oxidative stress, causing neuroinflammation and neurodegeneration, be reduced or reversed?

## Teaser

Neurological disorders, including Alzheimer’s disease, affect longevity and quality of life. Neurodegenerative pathologies share common features, like oxidative stress, closely related to inflammation, synapse dysfunctions, protein misfolding and defective autophagia. Antioxidant enzymes, like the manganese superoxide dismutase, can powerfully neutralize reactive oxygen species, decreasing neurological oxidative damage and inflammatory mechanisms.

## Graphical Abstract

**Graphical Abstract:**
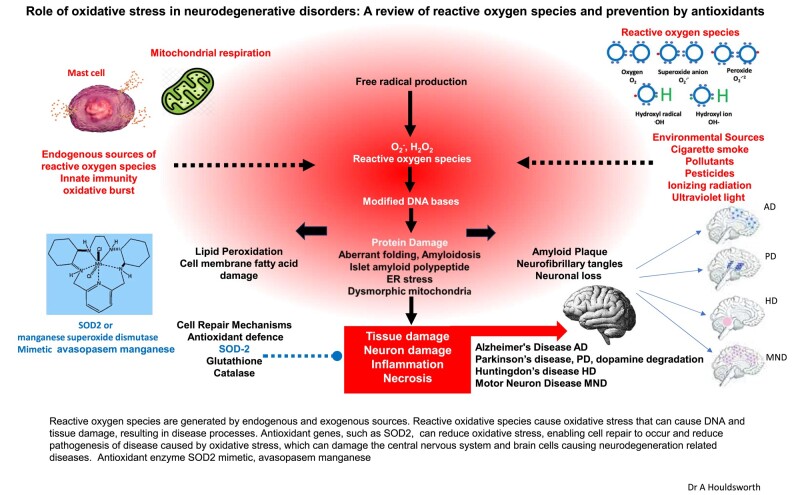

## Introduction

Globally, neurological disorders are the second leading cause of death and a leading cause of disability.^1^ The UK has 850 000 people living with incurable and difficult-to-treat forms of dementia, costing over 26 billion per year, predicted to double by 2040.^2^ Therefore, new approaches in the understanding and novel treatments for neurodegenerative disease are of great importance.

This review aims to examine some intracellular antioxidant mechanisms that reduce reactive oxygen species (ROS) and the effects of oxidative stress (OS) in the CNS and explores deficiencies of these mechanisms as a cause of disease in neurodegenerative disorders (ND), focusing particularly on Alzheimer’s disease, Parkinson’s disease and motor neuron disease. Multiple sources of research literature were explored, and the information was synthesized to provide an up-to-date overview of this topic.

The pathogenesis of the disease is largely caused by physiochemically altered proteins, because of the action of ROS. The focus of this review is to describe the action of antioxidant superoxide dismutase (SOD) in reducing OS. ND are characterized by neuronal damage due to OS, and some inflammatory processes and epigenetic factors that reduce antioxidant function are also discussed in the paper.

Although several drugs are prescribed for these disorders, some are not able to cross the blood–brain barrier (BBB), and their efficacy in treated ND is limited.^3^

## ROS, OS and neurodegenerative diseases

**Figure 1:**
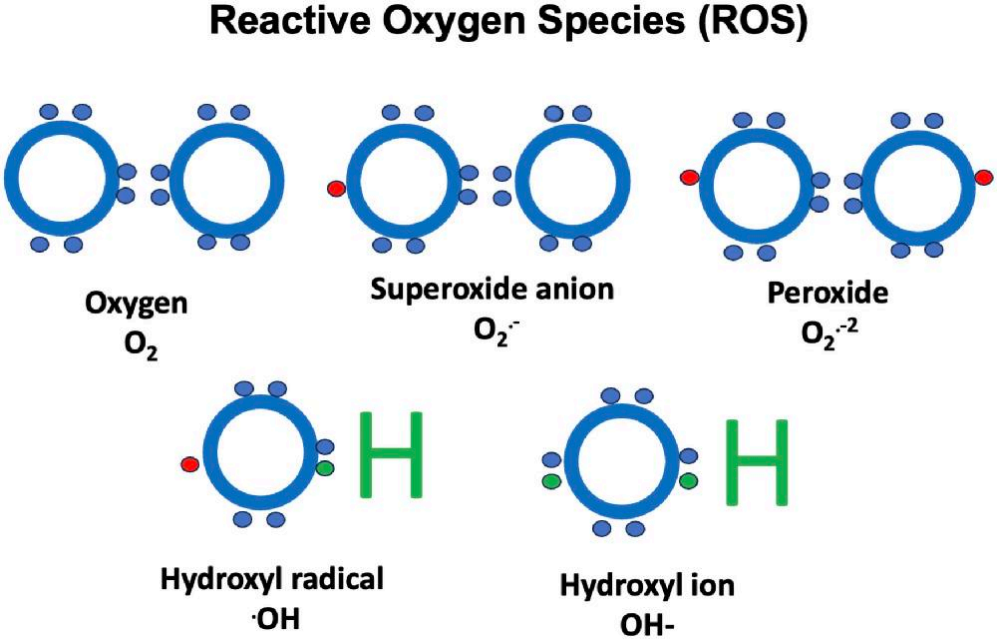
**ROS.** Examples of ROS as by-products of normal oxygen metabolism including peroxide, superoxide anion, hydroxyl radical and hydroxyl ion. All these species have an unpaired electron (except for the hydroxyl ion) making them highly unstable resulting in OS that can cause damage to cell structures and DNA. However, some low levels of ROS can have roles in intracellular signalling. Figure based on information from Phaniendra *et al*.^6^

ROS are groups of atoms that have odd, unpaired number of electrons, causing OS, and play a vital role in the pathophysiology of ND. This can be exacerbated by mitochondrial dysfunction or reduced antioxidant gene expression.^4^ There are several examples of free radicals (R•s) and these include superoxide, oxygen, hydroxyl, alkoxy and peroxyl radicals, as well as nitric oxide and nitrogen dioxide (Fig. 1).^5^ There are also non-radical ROS, such as hydrogen peroxide, hypochlorous acid and several nitrogen compounds. OS biomarker detection tools are available to investigate ND, such as immunofluorescence. Using nitro-tyrosine, 4-hydrononeal and 8-hydroxyguanine in hippocampal slices is an example of a method of detection.^7,8^ Other biomarkers that may be useful as diagnostic tools could be protein carbonyls.^8^

The free radical theory of aging is a well-established theory proposing that oxidative damage caused by ROS is a primary cause of aging.^9^ The effects of ROS, associated with ND, is well established in Alzheimer’s disease, Parkinson’s disease, Huntington’s disease, amyotrophic lateral sclerosis, multiple sclerosis, and Friedreich’s ataxia. Indeed, ROS have been shown to cause the methylation of genes altering the genetic epigenetic landscape. Many chronic diseases are triggered by environmental exposures, such as ROS, that can give rise to aberrant changes in the epigenome and can remodel DNA methylation in chronic disease.^9^

Genotypes of *APOE4* that have been strongly associated with OS in the brain have been recognized as susceptibility genes for Alzheimer’s disease phenotype and pathogenesis. ApoE4 is the least functional antioxidant of the ApoE family (ApoE1–4) in addressing OS, and its involvement in SOD2 antioxidant function is discussed later.^10^

**Figure 2:**
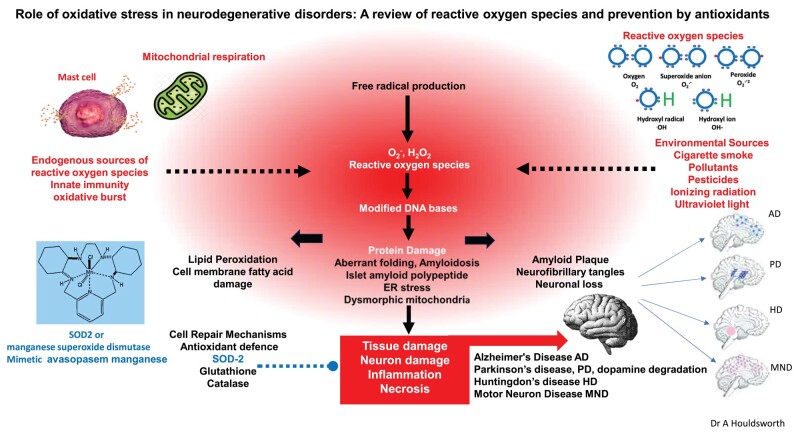
**OS and NDG.** ROS are generated by endogenous and exogenous sources. ROS cause OS that can cause DNA and tissue damage, resulting in disease processes. Antioxidant genes, such as SOD2, can reduce OS, enabling cell repair to occur and reduce pathogenesis of disease caused by OS. OS can damage the CNS and brain cells causing NDG-related diseases such as Alzheimer’s disease, Parkinson’s disease and motor neuron disease—these are indicated on the brain figures by the blue and purple areas of the brains, respectively. It is postulated that reducing OS may inhibit the pathogenesis of NDG. Figure based on information from Islam.^4^

Although there are both exogenous and endogenous sources, the main source of very active endogenous biological ROS is the mitochondria, where the superoxide anion radicals, hydrogen peroxide and hydroxyl radicals are generated during mitochondrial electron transport of four electron reduction of O_2_ to H_2_O_2_ (Fig. 2).^11,12^ Endogenous ROS are also generated through immune cell activation, inflammation, mental stress, excessive exercise, ischaemia, infection, cancer and aging,^6^ whereas exogenous sources include pollution, alcohol, tobacco, smoke, heavy metals, transition metals, industrial solvents, pesticides, radiation and some drugs.^8^

Reactions of an enzymatic nature include those generated by phagocytosis and the mitochondrial respiratory chain, which differ from non-enzymatic processes, such as oxidative phosphorylation in aerobic respiration. However, other minor sources of ROS are peroxisomes and endoplasmic reticulum with various catabolic pathways.^6,12^

These potentially dangerous reactive oxidative intermediates, as a by-product of normal metabolism, can play an important role in the pathogenesis of several disorders, including neurodegeneration (NDG).^13^ Both neural and vascular dysfunctions can also be mediated by the pseudohypoxia resulting from an increase in the cytosolic ratio of free NADH/NAD^+^, similarly to true hypoxia. An overproduction of ROS in the mitochondria can give rise to the oxidation of mitochondrial lipids, proteins and DNA. Further, it is known that OS has also been associated with the misfolding of proteins, as observed in Creutzfeldt–Jakob disease.^4^ However, modest increases in ROS can activate positive cellular responses, known as hormesis, and can counter normal aging processes compared to hyperactivation of signalling pathways that promote inflammation, cancer and cell death, as observed in the accelerated phenotype of aging.^9^ This is an example of where exposure to a low dose of a chemical agent or environmental factor is beneficial but damaging at higher doses.

Hormesis is an example of where exposure to a low dose of a chemical agent or environmental factor is beneficial but damaging at higher doses and where modest increases in ROS can activate positive cellular responses. Thus, although an imbalance of ROS can cause disease, these highly reactive species are also essential molecules with several physiological functions that sometimes act as second messengers in many tissues. Thus, ROS have a significant role as important signalling molecules in the nervous system, when present in low levels, but if the levels are elevated to disrupt their homeostasis, they also play a role in the progression of inflammatory disorders. When polymorphonucleocytes generate ROS at the site of inflammation during an immune response, it can result in tissue injury and the dysfunction of endothelia.^14^ There is evidence to suggest that ROS may influence multiple aspects of neural differentiation, where sub-lethal levels of R• species and hydrogen peroxide influenced intracellular signalling pathways. These mechanisms modulated the gene expression, cell differentiation and growth in several different neuronal and non-neuronal cells. One study suggests that normal ROS production regulates neuronal maturation in biochemistry, physiology and morphology and that some of these are processes are mediated by superoxide radicals, in particular.^15^ Microglia and astrocytes can be activated by these reactive species.^16^

It must also be noted that the generation of mitochondrial ROS is valuable bactericidal ammunition in the innate immune system during bacterial, viral and fungal infections.^12^ These highly ROS are indiscriminate in their targeting, thus causing damage to local tissue cells besides pathogens.^13^ This production of ROS during infection, as the immune response is triggered, can damage healthy cells and induce chronic inflammation, including neuroinflammation (NI) and degeneration.^12,16^

Inflammasome activation, due to OS, is associated with multiprotein complexes that accumulate in the cytosol and lead to inflammatory processes with dysfunctional cell clearance.^17^

Another source of OS in some areas of the brain that contain high amounts of iron can stimulate R• reactions, such as the superoxide anion and hydrogen peroxide. There is an age-related build-up of iron complexes and may be a potential biomarker tool for diagnosing ND.^18^ The accumulation of iron in the brain requires strict regulation to prevent it from generating ROS, which can affect DNA expression through epigenetic mechanisms.^18,19^

## Antioxidants

Antioxidants, in general, are natural polyhydroxylated phenolic compounds, with low molecular weights. Some cellular enzymes are expressed within cellular compartments with powerful antioxidant properties that eliminate R•s.^20^ Many vegetables and fruits also contain dietary polyphenols with antioxidant properties, including flavonoids, phenolic acids, tannins, lignans, stilbenes, catechins and carotenoids.^21-23^ It is thus clear that antioxidants prevent the intracellular oxidation of molecules. Electrons or hydrogens are removed from a substance in this process, and thus, they can decrease the oxidative damage to a cell by reacting directly with R•s. It is considered that the location and number of hydroxyl groups on aromatic rings of these antioxidant substances may play important roles in antioxidant activity.^20^ Thus, antioxidants are R• scavengers that can decrease the oxidative damage caused by ROS. Neutralization of ROS by antioxidants can be endogenous or exogenous in origin.^24,25^

## Superoxide dismutase

**Figure 3:**
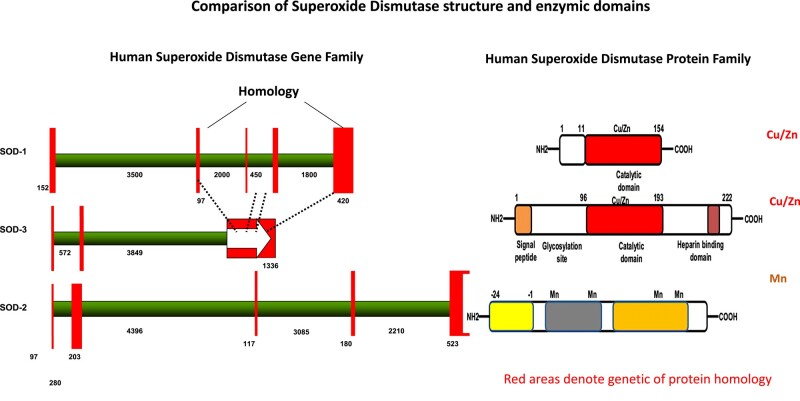
**SOD genes.** A comparison of the different SOD genes and the enzymes that they encode, showing the catalytic domains that bind Cu/Zn in SOD1 and SOD3 and Mn in SOD2. Homology is shown between intracellular SOD1 and extracellular SOD3. There is no significant amino acid homology between SOD2 and SOD1 and SOD3. Figure adapted from Sah *et al*.^11^

**Figure 4:**
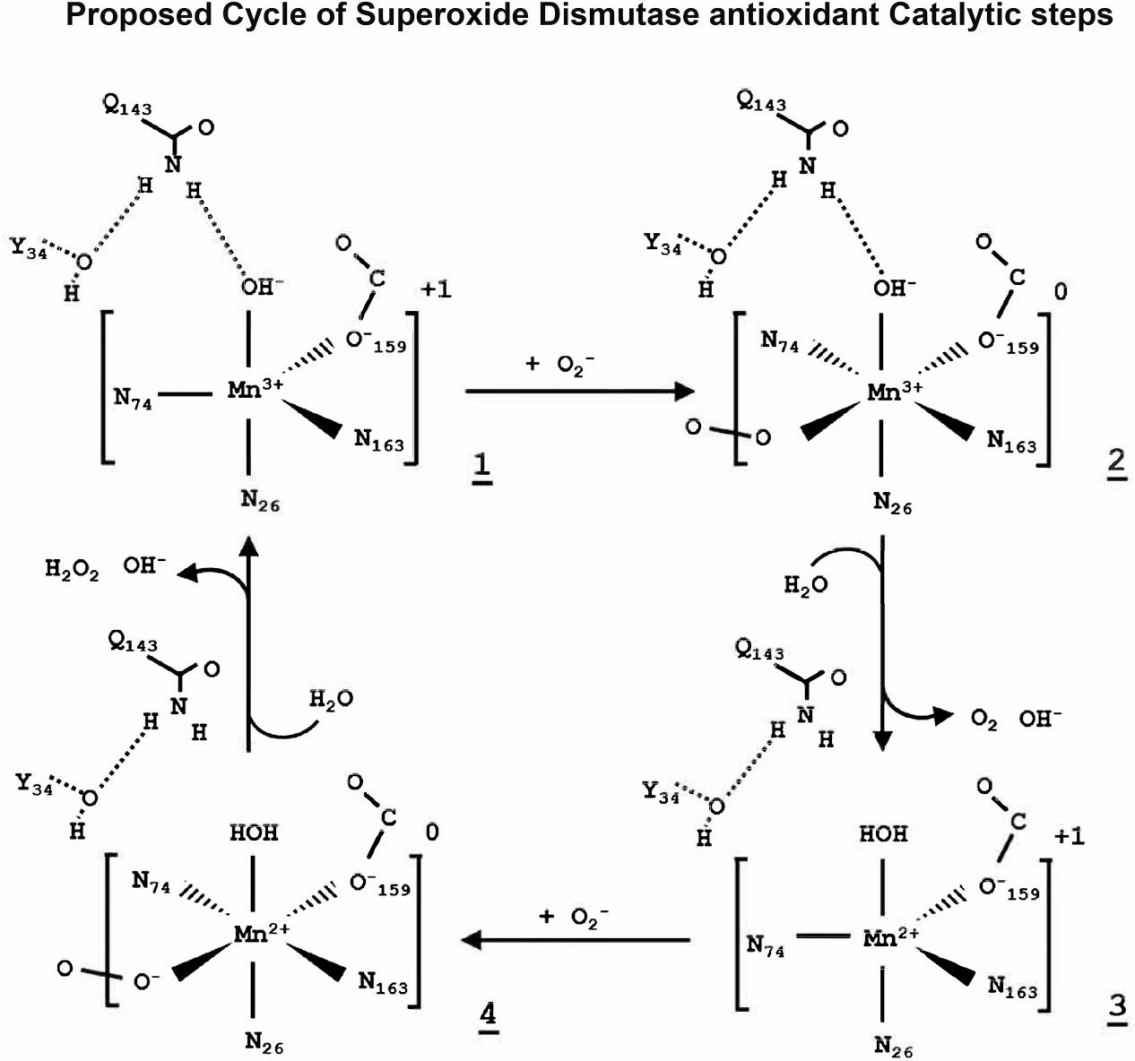
**Antioxidant mechanism of SOD2.** Proposed mechanism for Mn SOD (SOD2) chemical reaction in neutralizing superoxide ROS. Figure adapted from Azadmanesh and Borgstahl^28^ and Azadmanesh *et al*.^29^

The ubiquitous family of SOD enzymes that catalyze the dismutation of superoxide anions can be associated with copper, manganese (Mn), zinc and iron, depending on their function, where copper/zinc-associated SOD1 is active in the cytosol and organelles. SOD3 is an extracellular enzyme, and SOD2 is active in mitochondria, with several structural differences (Fig. 3).^26^ The gene for SOD2 enzyme is found on Chromosome 6, and abnormalities of Chromosome 6 have been associated with Alzheimer’s disease, indeed with 120 additional disorders.^27^ SOD2 has a metalloenzyme antioxidant activity and is dependent on the transition metal Mn, which is inserted into the complex when SOD2 is newly synthesized. SOD2 neutralizes superoxide radical and is converted into hydrogen peroxide,^26^ controlling dioxygen toxicity in the mitochondria, an organelle of extreme oxidative load (Fig. 4).^28,29^ Mn is a highly studied transition metal, and significantly reduced levels of Mn have been measured in patients with Alzheimer’s disease and associated with mild cognitive impairment.^30^ There are other key enzymes than SOD2 dependent on Mn, such as glutamine synthetase, arginase and pyruvate carboxylase.^31^

Several pathological phenotypes are associated with a loss of SOD2 activity.^32^ There is early lethality in complete *SOD2^−/−^* knockout mice, but the effects of antioxidant dysfunction have been shown in *SOD2^+/−^* mice experiments, where animals with low antioxidant capacity experienced direct damage to lipids, proteins and DNA, though OS also caused behavioural impairments as well as increased OS makers in the hypothalamus and cortex.^33^ Indeed, overexpression of *SOD2* in mice reduces hippocampal superoxide and prevents memory deficits.^34^ It is postulated that employing Cre-Lox technology, thus enabling neurons to be targeted specifically to experimentally reduced *SOD2* expression in the nervous system, may be a way of further investigating the direct effects of SOD2 deficiency.^33^

In humans, there is evidence of age-related reduction of antioxidant gene expression, observed in the reduced expression of mRNAs coding for SOD1, SOD2 and catalase in the preovulatory follicles of women over the age of 38 years, indicating an age-related reduced defence against ROS.^35^

*SOD2* expression can also be affected by genetic variation in the gene. Some polymorphisms of the *SOD2* gene, such as V16A polymorphism (rs4880), have a reduced expression compared to the *SOD2* wild type and are known as a susceptibility gene for various conditions emanating from OS. There is an association of the *SOD2* C47T polymorphism with mild cognitive impairment associated with Alzheimer’s disease in carriers of the *APOE4* allele.^36^ There are some inconsistencies in these findings when associating *SOD2* genotypes with Alzheimer’s disease, as some researchers consider that *SOD2* (rs4880) does not have a determining role in Alzheimer’s disease in the same way that *SOD1* has in Parkinson’s disease.^37^ However, the role of the *SOD2* gene in reducing OS and neutralizing ROS remains important in the pathogenesis of oxidative damage and some diseases.

Another antioxidant that was shown to ameliorate OS and increases *SOD2* expression is melatonin. When administered to irradiated tissue, melatonin boosted SOD2 activity in normal tissue but not cancer tissue.^35^ The hypothalamic–pituitary–adrenal gland axis has a stress-induced increase of cortisol levels that can cross the BBB, which can also induce ROS and OS and reduce the expression of melatonin, which may affect SOD2 activity. Loss of the circadian rhythm of cortisol, with a flatlining of high cortisol levels, particularly at night, can reduce melatonin activity.^38^ Melatonin is essential for sleep healthy patterns, and reduced sleep is associated with reduced memory retention.^39^ Could this further reduce SOD2 activity and further contribute to neurodegenerative damage? In addition to antioxidant replacement, melatonin supplementation can have a positive effect on antioxidant activity.^40^

Cadmium has been found to inhibit antioxidant properties of enzymes, such as SOD2, and catalase.^41,42^ It also causes mutations and chromosomal deletions while also enhancing the production of ROS. A high source of exogenous cadmium in humans is from smoking cigarettes, and it is reported that 30% of smokers develop vascular dementia and 40% develop Alzheimer’s disease.^39,41^

## SOD2 as a pharmacological agent

Currently, there are few examples of SOD2 administration in clinical practice. In considering the concept of SOD2 therapy, the exogenous SOD2 supplementation by oral delivery may be complicated by the formation of anti-drug antibodies to the exogenous protein or digestion of the protein before it is absorbed, so new methods of delivery are under investigation. Intramuscular delivery of SOD2 is a possibility. In addition to supplementation of SOD2, demethylation of the silenced gene or enhanced transcription through associated transcription factors has been suggested.^43^ One bovine source of SOD, called orgotein, can be administered intramuscularly but can contain some contaminants that cause hypersensitivity.^44^ Recent developments in pharmaceutical advancements have produced avasopasem Mn (GC4419), a SOD mimetic, that can selectively reduce superoxide anions to hydrogen peroxide molecules. The drug has been developed by Galeria. Trials have been carried out administering avasopasem Mn as a treatment for oral mucositis and esophagitis after radiation therapy, and the drug appears to be well tolerated and found to protect normal tissues; the drug was administered by infusion in the clinical trials.^45^

## Neurodegeneration

Age-related and progressive NDG involves ataxia and dementia, affecting the longevity and quality of those affected by these disorders. Neurons and glial cells are more susceptible to OS and have a higher metabolic demand. This, combined with the lower rate of regeneration compared to other cells in the body, with inadequate antioxidant potential makes the CNS vulnerable to oxidative damage.^46^ Thus, poor antioxidant status or an imbalance of oxidant and antioxidant equilibrium is implicated in the pathogenesis of ND.^32^ Brain tissue is highly metabolically active, depending on oxidative phosphorylation for its energy source,^47^ and has high levels of lipids, which also consume high levels of oxygen. Thus, OS can affect the function of neurons and their survival. A broad spectrum of ND is associated with this kind of antioxidant dysregulation.^32^

Mitochondrial proteostasis genes that regulate the chaperoning, folding and maintenance of protein function are downregulated, and protein damage that is not reparable accumulates with age.^48,49^ The acute imbalance between the accumulation of ROS and antioxidant defence mechanisms results in OS, as previously described, and this is thought to have a principal role in the pathogenesis of Alzheimer’s disease, where imbalance promotes the expression of mitochondrial genes involved in metabolism and the ROS generated causes gene mutations in mitochondrial DNA.^48,50^

NI is a cascade of reactions and immune events, resulting in NDG, and some evidence is developing that OS and NI both contribute to the pathophysiology, onset and progression of NDG. OS can contribute to neurodysregulation and inflammation.^46,50^ As discussed in the following section, mast cell (MC) activation involves the degranulation of cytoplasmic granules containing inflammatory mediators, some of which are ROS contributing to OS^51^ (Fig. 1).^29^

NDG can be characterized by a specific population of neurons becoming progressively dysfunctional due to aberrant conformations of microtubule-related protein tau where tau is a protein that stabilizes the internal skeleton of neurons. Tauopathies are associated with many neurodegenerative diseases where the most presented is Alzheimer’s disease. OS is a significant factor in the pathophysiology of neurodegenerative tauopathies. Tau hyperphosphorylation is associated with the formation of insoluble aggregates of neurofibrillary tangles, synaptic dysfunction and neuronal death, and this can be induced by OS because it promotes the phosphorylation of the protein, thus decreasing the binding affinity of tau towards microtubules. Mitochondrial OS causes hyperphosphorylation of tau, and this can be reduced by antioxidants. SOD2-deficient mice experience a greater hyperphosphorylation of tau than the wild type.^52,53^

## Immunology of NDG

The CNS is a site of immune privilege, and peripheral immune cells are not able to cross the BBB, but unlike many other haematopoietic immune cells, MCs are present in the human brain and found in several structures that mediate sensory or neuroendocrine areas while interacting with the neuroimmune system.^54^

Whereas MCs are the first responders during a pathogen invasion, activated microglia cells and the presence of certain cytokines are reported to be key players in NI.^50,54,55^ MCs act as catalysts that initiate and amplify the immune and nervous response, recruiting other inflammatory factors.^51^ They are also present in the blood–CSF barrier and in the dural layer of the meninges.^51^ In the brain, they are effectors and sensors on the brain side of the BBB where they interact with microglia and astrocytes. NI is one mechanism that leads to the progression of NDG, dysfunction and neuronal loss.

Corticotropin-releasing hormone is released during stress-related NDG, released by MCs contributing to the hypophysial–pituitary–adrenal axis. Thus, MCs interact between the immune system and neurons. Releasing this hormone is also a response to stress and induces degranulation of MCs, which can disrupt the BBB and lead to ND.^55^

These inflammatory mechanisms in the brain can drive formation of amyloid and Aβ plaques that enhance the onset of dementia and Alzheimer’s disease; tau forms synaptotoxin aggregates that develop into neurofibrillary tangles.^56^

As first responders in the CNS, MCs degranulate inflammatory mediators that form a chemotactic pathway for glial cells towards the pathogenic stimuli.^57^

Microglia are innate immune cells of the CNS and have an essential role to play in NI.^58-60^ Microglia are scavengers of neural dead cells and are believed to be involved in brain defence. They are an important part of the neural immune system and are largely involved in the clearance of amyloid-beta and the development of NI. This important role plays a part in the pathogenesis of Alzheimer’s disease. In addition to causing the neuronal damage that leads to the pathogenesis of Alzheimer’s disease, microglia are also suggested to form a protective barrier surrounding amyloid deposits, where amyloid fibrils are compacted, and microglia act as housekeeping phagocytes, thus maintaining homeostasis by Aβ facilitating removal.^61^ Microglia may also be activated through an excessive generation of superoxide radicals.^60^ However, antioxidant status of the tissue must be adequate to address this increased OS.^62^

Although some researchers observe that microglia engulf plaques and decrease the pathology, it is also thought that they may be responsible for spreading the pathology, where activation of microglia can contribute to NI, releasing inflammatory mediators, including ROS. Two distinct forms of microglia are identified, homeostatic and disease-associated microglia phenotypes.^63,64^ Some new therapies involving the modulation of microglia phagocytosis have been suggested.^65^

## Parkinson’s disease

In the case of Parkinson’s disease, the degeneration of dopaminergic neurons involves OS. When physiologic maintenance of neuron redox potential is disrupted by biological processes, this can lead to cell death.^66^ Both oxidative and nitrative damage have been recognized in Parkinson’s disease substantia nigra. Dopaminergic neurons in the substantia nigra are diminished in Parkinson’s disease. Dopamine is an unstable molecule that causes the generation of ROS and dopamine quinones, in this region of the brain, causing OS, through auto-oxidation.^41^ This OS and mitochondrial dysfunction are central elements in the NDG in Parkinson’s disease.^67^ Aging reduces the ability to maintain antioxidant mechanisms against OS, through reduced activity and expression of antioxidant genes, such as SOD2, catalase and glutathione peroxidase.^9,35^

Dopamine itself undergoes auto-oxidation and is an unstable molecule forming quinones and R•s.^68,69^ SOD2 enzyme activity is modified by dopamine quinones and has implications for Parkinson’s disease.^67^

When the brain cells in the substantia nigra that express dopamine are lost, this causes Parkinson’s disease.^68^ Dopaminergic neurons in the midbrain posterior to the crus cerebri fibres of the cerebral peduncle, pars compacta part of the substantia nigra, are responsible for dopamine production. Basal ganglia nerve cells control movement, and when they die or are impaired, they reduce electrical signals to the body.^69^

A *SOD2* V16A polymorphism (rs4880) has been associated with increased susceptibility to Parkinson’s disease in a Chinese population.^70^ This V16A polymorphism has a lower expression of Mn SOD than the wild version of the gene and has been shown to be associated with other disease states, such as diabetic nephropathy.^67^

Administering functional *SOD2* may be beneficial to Parkinson’s disease patients in reducing the OS immunopathology associated with the NG of these patients.

## Motor neuron disease

There are two recognized forms of motor neuron disease in humans, one familial and one acquired. The familial version of the disease is associated with several genes but predominantly *SOD1* mutations. SOD1 is an extracellular antioxidant that reduces the R• oxygen species to hydrogen peroxide. The familial form of the disease appears in about 12–20% inherited gene defects and 1–2% are spontaneous mutations of the aberrant SOD1 protein and are sporadic in nature.^71^

Recent approaches to treatment are to neutralize the dysfunctional amyotrophic lateral sclerosis gene product of SOD1 with a monoclonal antibody directed at the protein, called tofersen (BIIB067).

New therapies were trialled using m-RNA to block the aberrant SOD1 protein from being functional, and remarkable improvement in the progress and severity of the disease has been observed. Clinical benefits of a Phase 3 trial (VALOR trial) for patients with the SOD1 mutation were reported on in 2021.^72,73^

As previously mentioned, the accumulation of iron in brain tissue generates ROS and requires strict regulation, which may be generated from dysfunctional SOD1 enzymes.^74^

Different to SOD2, the bio-metallic enzyme, SOD1, relies on iron or copper to function as a molecule.^26^

The antioxidant function of these enzymes is essential in reducing the OS caused by ROS in the body, thus inhibiting the production of aberrant proteins that cause the damage to neurons. In SOD2-deficient mice, there are age-related motor neuron disease–associated signalling alterations.^75^  *SOD2* knockout mice have early lethality and NDG but Cre-Lox technology enables SOD2 to be reduced in neurons.^33^

Thus, could the expression of the non-functional or aberrant form of SOD1 and SOD2 be corrected to reduce ROS and its associated pathology in some patients?, Or could the reinstatement with exogenous functional SOD1 further enhance current treatment for familial amyotrophic lateral sclerosis patients and the administration of SOD2 therapy may reduce OS in motor neuron disease generally?

## Alzheimer’s disease

The pathogenesis of Alzheimer’s disease is considered to be the result of an excessive generation of ROS causing neurofibrillary tangles as aggregates of tau, in addition to amyloid plaque aggregation of Aβ. This can also give rise to higher metabolic demand and/or mitochondrial dysfunction.

As referred to earlier, the hallmarks of Alzheimer’s disease include tau and amyloid deposition and in one study tau increase of SOD1 expression, which in animal studies, the wild-type *SOD1* gene expression was found to be protective against NDG.^52^ It is well understood that familial amyotrophic lateral sclerosis is due to a mutation of SOD1 (Cu/Zn).^76^ Indeed, several of the chronic neurodegenerative pathologies of the CNS share some common features, such as inflammation, OS, synapse dysfunctions, protein misfolding and defective autophagia.^4,9^

As previously explained, the brain produces large amounts of ROS, as it is a highly metabolically active organ, usually protected by an elaborate network of antioxidants that maintain the delicate equilibrium; thus, OS is thought to contribute to Alzheimer’s disease pathogenesis.^62^

Also mentioned earlier, Mn status is reduced in some Alzheimer’s disease patients, and SOD2 antioxidant function is dependent on this transition metal,^30^ thus may be supportive evidence that SOD2 function may be a significant factor in the pathogenesis of Alzheimer’s disease.

## Epigenetic landscape and effects of aging on antioxidant mechanisms

Epigenetic changes alter the accessibility to genetic material and expression of proteins, by causing genes to be inappropriately silenced through methylation of DNA. Histone can also undergo modification by non-coding RNA, otherwise described as junk RNA, and can be altered epigenetically.^77^ Methylomic changes have been noted in a few loci in post-mortem brain samples of Alzheimer’s disease patients with psychosis. Hypermethylation is observed with age and can be identified by biochemical tests as biomarkers for the epigenetic clock, where geroscience is attempting to measure the phenotypic age of patients through these markers.^78^ As a result of these changes of the age-related epigenetic landscape, where some are a result of reactive oxidative damage to DNA, the accumulation of OS can cause cognitive aging and NDG. SOD2 has been shown to be affected and modified by such mechanisms, especially because of diabetes.^67,76^ Hypermethylation of CpG nucleotides within the *SOD2* promoter has been associated with reduced SOD2 expression.^79^

*APOE4*, as previously mentioned, is a susceptibility gene for Alzheimer’s disease that has antioxidant activity. In an experiment demonstrating the actions of antioxidant function, APOE4-targeted replacement mice had a higher expression of antioxidant activity against OS.^10^ If the expression of other antioxidants, such as *SOD2*, is also silenced or their expression diminished due to age, epigenetic alterations could explain the increased OS associated with Alzheimer’s disease pathogenesis.^10^ Further, although there is some discussion about the direct association of rs4880-T allele of the SOD2 gene with Alzheimer’s disease, patients with mild cognitive impairment and Alzheimer’s disease are more prevalent in individuals with a combination of the associated *APOEe4* allele and *SOD2* polymorphism.^30,36^

## Conclusion

It is clear from the research that an imbalance of ROS generation can be a cause of OS in neural tissue, but also effective antioxidant function can reduce the resultant oxidative damage from ROS (Table 1).^25,80^ Antioxidant mechanisms clearly have the potential to reduce OS by eliminating ROS; however, antioxidant gene expression can be affected by age and epigenetic events as well as DNA damage mechanisms, reducing their ability to reduce ROS. In addition, sleep deprivation may diminish the action of melatonin, an activation factor for SOD2.

**Table 1:** ND, for example, of different pathogenesis of cell deregulation, aberrant proteins and genetic associations, highlighting the different areas of the brain that are affected in the disorders adapted from Bernaus *et al.*^80^ and Li *et al.*^81^

	Neurodegenerative disorder			
	Alzheimer’s disease	Parkinson’s disease	Huntington’s disease	Motor neuron disease (amyotrophic lateral sclerosis)
**Agent**	OS	OS	OS	OS
**Glial deregulation**	Phagocytic clearance, NI, autophagy, microgliosis, BBB dysregulation			
**Epigenetic factors**	DNA methylation, chromatin remodelling and histone post-translational modifications			
**Mode of action**	Activation of macromolecule, glial cell and tau phosphorylation	Lipid peroxidation and mutation in α-synuclein	Lipid peroxidation	SOD1 activation, inflammation Familial form has *SOD1* mutation
**Aberrant protein and cause of neuronal deregulation**	Aβ plaques, tau neurofibril tangles	α-Synuclein misfolding protein	Huntingtin (Htt) protein	Multiple impaired protein homeostasis due to ER stress
**Gene association**	*ApoE4*, amyloid precursor protein (*APP*), presenilin 1/2 (*PSEN1/2*)	*SNCA*, *LRRK2*	*HTT* (*IT15*)	*SOD1*, *TDP-43*, *FUS*
**Area of the brain affected**	Hippocampus	Basal ganglia and substantia nigra	Caudate nucleus, putamen and globus pallidus	Lower and upper motor

After presenting all this evidence, questions about changing the microenvironment of the CNS must be considered, before irreversible damage occurs, regarding pathogenic levels of ROS and OS, and can any of this damage be reversed.^81^ It is hoped that this review will contribute to the knowledge and understanding of preclinical research for the enhancement of clinical translational therapies for patients with diseases caused by OS-induced NDG.

Advancements in technology could improve strategies to address OS-induced disease, and the recent era of nanotechnology offers new frontiers of opportunity for administering drugs to cross the BBB, which mainly involves the opening of tight junctions and the inhibition of efflux pumps.^3^

As well as antioxidant mimetics, methods to increase the activity of antioxidant enzymes may reduce the oxidative damage to neural tissue, as observed in Alzheimer’s disease, Parkinson’s disease and motor neuron disease.

Increased expression of *SOD2* and mimetic alternatives with similar function have been shown to reduce ROS and protect against OS in clinical trials for other causes of OS, such as post chemotherapy radiotherapy–induced oxidative damage in irradiated tissue. New mimetic drugs that mimic the activity of SOD2 have been successful in reducing damage from radiotherapy-induced OS and may also be able to reduce the progression of NDG. It is hoped that clinical trials to test this hypothesis, by enhancing antioxidant function, may be effective against some ND.

Early diagnosis of ND diseases is improving, especially with the discovery of susceptibility genes and different combinations of gene expression observed in patients with Alzheimer’s disease, Parkinson’s disease and motor neuron disease. It is hopeful that a new era of early diagnosis and the reduction of OS may reduce the pathogenesis of some ND at an early stage of disease.

Some suggestions for increasing our understanding of this topic include establishing the antioxidative status of patients with Alzheimer’s disease, genotyping associated gene polymorphisms, measuring antioxidant status in patients and testing patients for their Mn, Cu and Zn status as well as OS markers.

A clinical trial of SOD2 mimetics for some patients with ND would be an interesting step forward in assessing its efficacy in patient at an early stage of their diagnosis.

## Data Availability

Data sharing is not applicable to this article as no new data were created or analysed in this study.
